# A Select Subset of Electron Transport Chain Genes Associated with Optic Atrophy Link Mitochondria to Axon Regeneration in *Caenorhabditis elegans*

**DOI:** 10.3389/fnins.2017.00263

**Published:** 2017-05-10

**Authors:** Wendy M. Knowlton, Thomas Hubert, Zilu Wu, Andrew D. Chisholm, Yishi Jin

**Affiliations:** ^1^Section of Neurobiology, Division of Biological Sciences, University of CaliforniaSan Diego, CA, USA; ^2^Howard Hughes Medical Institute, University of CaliforniaSan Diego, CA, USA; ^3^Department of Cellular and Molecular Medicine, School of Medicine, University of CaliforniaSan Diego, CA, USA

**Keywords:** electron transport chain, growth cone, oxidoreductase *rad-8*, iron-sulfur protein, mitochondrial unfolded protein response

## Abstract

The role of mitochondria within injured neurons is an area of active interest since these organelles are vital for the production of cellular energy in the form of ATP. Using mechanosensory neurons of the nematode *Caenorhabditis elegans* to test regeneration after neuronal injury *in vivo*, we surveyed genes related to mitochondrial function for effects on axon regrowth after laser axotomy. Genes involved in mitochondrial transport, calcium uptake, mitophagy, or fission and fusion were largely dispensable for axon regrowth, with the exception of *eat-3/Opa1*. Surprisingly, many genes encoding components of the electron transport chain were dispensable for regrowth, except for the iron-sulfur proteins *gas-1, nduf-2.2, nduf-7*, and *isp-1*, and the putative oxidoreductase *rad-8*. In these mutants, axonal development was essentially normal and axons responded normally to injury by forming regenerative growth cones, but were impaired in subsequent axon extension. Overexpression of *nduf-2.2* or *isp-1* was sufficient to enhance regrowth, suggesting that mitochondrial function is rate-limiting in axon regeneration. Moreover, loss of function in *isp-1* reduced the enhanced regeneration caused by either a gain-of-function mutation in the calcium channel EGL-19 or overexpression of the MAP kinase DLK-1. While the cellular function of RAD-8 remains unclear, our genetic analyses place *rad-8* in the same pathway as other electron transport genes in axon regeneration. Unexpectedly, *rad-8* regrowth defects were suppressed by altered function in the ubiquinone biosynthesis gene *clk-1*. Furthermore, we found that inhibition of the mitochondrial unfolded protein response via deletion of *atfs-1* suppressed the defective regrowth in *nduf-2.2* mutants. Together, our data indicate that while axon regeneration is not significantly affected by general dysfunction of cellular respiration, it is sensitive to the proper functioning of a select subset of electron transport chain genes, or to the cellular adaptations used by neurons under conditions of injury.

## Introduction

Most neurons are intrinsically competent to regenerate their axonal processes after damage, although neurons of the adult mammalian central nervous system are generally unsuccessful in their regrowth attempts (He and Jin, 2016). Extensive studies in multiple model systems have revealed a complex set of intrinsic and extrinsic regulators of axon regeneration, and efforts are underway to understand these mechanisms with the aim of coaxing neurons into regrowing their connections and restoring neural function (Tedeschi and Bradke, 2016).

Successful axon regeneration is an intricate multistep process: The initial injury generates various cellular signals that must be detected, propagated, and interpreted by the neuron, and early responses include resealing the disrupted membrane and stabilizing damaged structures. Following this, the “repair” response involves preparing cellular structures for repair, initiating genetic growth/regrowth programs, synthesizing cellular components needed for regeneration and transporting them to the injury site, and the formation of pro-growth structures such as growth cones. Finally, for functional restoration of the neural circuit, regrowing axons must reach their targets, pathfinding successfully in the post-developmental environment while overcoming growth-inhibiting factors.

Using the nematode *Caenorhabditis elegans*, we and others have taken a genetic approach to identifying molecular mechanisms of axon regeneration, reviewed in Chisholm et al. (2016). The axons of *C. elegans* sensory and motor neurons respond to damage by forming growth cones at the severed axon stump followed by extending the axon and eventually reconnecting to targets (Yanik et al., 2004). As in other animals, axonal injury triggers an initial transient change in axonal calcium levels, the dynamics of which are important determinants of subsequent regrowth (Ghosh-Roy et al., 2010). A MAP kinase cascade involving the dual leucine-zipper kinase DLK-1 is essential and rate-limiting for early steps in regeneration, including growth cone formation (Hammarlund et al., 2009; Yan et al., 2009; Yan and Jin, 2012). DLK-1 activity is regulated by calcium and acts as a link between initial injury signals and subsequent cytoskeletal and transcriptional responses (Ghosh-Roy et al., 2012; Chen et al., 2015). In response to damage, the axonal microtubule cytoskeleton undergoes an intricate sequence of changes resulting in the formation of a regenerative growth cone between 4 and 6 h after axotomy (Ghosh-Roy et al., 2012; Chen et al., 2015). Subsequent axon extension over the next 48 h is characterized by erratic guidance, frequent branching and pruning, yet can result in functional reconnection with the original targets (Yanik et al., 2004; Ghosh-Roy et al., 2010).

In our previous screen of more than 650 genes with human homologs, two genes in the mitochondrial electron transport chain (ETC), *isp-1* and *nduf-2.2*, were found to be required for axon regeneration in peripheral lateral mechanosensory (PLM) neurons (Chen et al., 2011). The *isp-1* gene encodes the sole iron-sulfur protein in the ubiquinol-cytochrome c reductase complex, or Complex III, of the ETC (Feng et al., 2001). The *nduf-2.2* gene encodes one of seven iron-sulfur proteins in the NADH ubiquinone oxidoreductase complex, also known as ETC Complex I (Kayser et al., 2001). Loss of function in either gene had no obvious effect on neuronal development but significantly reduced PLM axon regeneration, suggesting that axon regrowth depends on mitochondrial ETC function.

To further explore the contribution of mitochondria to axon regrowth, we have conducted a targeted screen of viable mutants defective in mitochondrial regulation or function. From this screen we have identified two additional Complex I iron-sulfur protein genes, *gas-1* and *nduf*-7, as well as the putative ETC component *rad-8* as being required for effective regeneration. Interestingly, loss of function mutants of most ETC component-encoding genes as well as mutants in genes relating to mitochondrial transport, calcium uptake, mitophagy, or mitochondrial fission/fusion did not affect axon regrowth, with the exception of the mitochondrial inner membrane fusion-promoting gene *eat-3*. Most of the axon regeneration-defective mutants responded to injury by forming growth cones at a normal rate, but exhibited decreased axon regrowth. Overexpression of either *isp-1* or *nduf-2.2* in the nervous system was sufficient to enhance axon regrowth beyond that seen in controls. Genetic double mutant analysis suggested that mitochondria act downstream or in parallel to injury-related calcium or DLK-1 signals. Our genetic analyses support a role for *rad-8* in the mitochondrial ETC, and revealed an unexpected genetic interaction between *rad-8* and the demethoxyubiquinone dehydroxylase *clk-1*, which synthesizes ubiquinone for use in the ETC. Additionally, mutants of *atfs-1*, a transcription factor involved in the mitochondrial unfolded protein response, suppressed defective regrowth seen in the *nduf-2.2* mutant despite having normal axon regeneration levels on their own. Together our data reveal a role for mitochondrial function in the extension of regrowing axons after injury, although it may not be the proper functioning of the ETC *per se* that determines regeneration success, but rather the cellular adaptations in injured neurons.

## Materials and methods

### Genetics and strains

*C. elegans* were cultured on nematode growth medium plates seeded with OP50 *E. coli* at 20°C for all experiments. Most strains contained P*mec-4*-GFP*(zdIs5)* for visualization of touch neurons, except those used for axotomy with the mito-GFP marker, which contained the P*mec-4-TagRFP(juIs252)* transgene. Mutants were obtained from Shohei Mitani's lab through the Japan National Bio-Resource Project, or from the *Caenorhabditis* Genetics Center, which is funded by NIH Office of Research Infrastructure Programs (P40 OD010440). All mutations were outcrossed at least twice to wild type. Alleles and strains used, as well as primer sequences for genotyping, are listed in Table S1.

### Molecular biology and transgenes

For rescue and overexpression experiments, coding sequences were cloned from N2 wild type genomic DNA using primers listed in Table S2 into the pCR8 backbone (Invitrogen) to create Gateway entry clones, which were then recombined into Gateway destination vectors to generate expression plasmids. For the mitochondrial GFP marker, the N-terminal 29 amino acid mitochondrial targeting sequence of human COX8a was cloned upstream of GFP using Gibson Assembly (New England Biolabs) into pCR8 to generate a Gateway entry vector, and then recombined with tissue-specific Gateway destination vectors.

Transgenic arrays were generated following standard microinjection procedure (Mello et al., 1991), with expression plasmids at the concentrations listed in Table S3. RFP or mKate2 driven by promoters for AIY neurons (*ttx-3*), AFD neurons (*gcy-8*), or coelomocytes (*unc-122*) was used as visual markers for transgenic arrays. Most arrays were created in wild type worms and crossed into the mutant backgrounds using primers listed in Table S4 to distinguish array sequences from genomic loci.

### Laser axotomy and microscopy

Final larval stage worms (L4) were anesthetized in M9 buffer containing 0.1% phenoxypropanol and mounted on agar containing 0.03% phenoxypropanol. PLM femtosecond laser axotomy and regrowth quantitation were performed essentially as described (Wu et al., 2007). For the experiment using alternate anesthesia, 0.1% levamisole in M9 buffer was used at both time points, and the agar for mounting did not contain any drugs. Axon regrowth measurements were obtained from three-dimensional reconstructions of ~1 μm sections using the Zeiss LSM Image Browser software, and any neurite >1 μm was included in the analysis. Strains were tested in at least two separate experiments and any sick animals or animals with regrowing axons fused to the severed neurite fragment were censored from the analysis.

For mitoGFP quantification, worms were mounted in phenoxypropanol as above and visualized at 63x using an LSM710 confocal microscope (Zeiss). For quantification of axonal mitochondrial density without injury, discrete axonal GFP puncta were quantified from 0 to 170 μm from the PLM cell body. For mitoGFP density measurements after injury, the number of discrete axonal GFP puncta were counted in the entire segment of the axon still attached to the cell body at both the time of injury (0 h) and 24 h later.

### Statistical analyses

Data are graphed as mean ± SEM using GraphPad Prism (version 5.01). All data were tested for statistical significance using unpaired Student's *t*-tests or *ANOVA* with Tukey's multiple comparison post-test in comparison to wild type animals tested on the same day. ns = *p* > 0.05, ^*^p < 0.05, ^**^p < 0.01, and ^***^p < 0.001. Numbers in graphs are the number of animals tested.

## Results

### Regrowing PLM axons maintain mitochondrial density

Mitochondria in *C. elegans* neurons have been visualized using mito-GFP markers (Fatouros et al., 2012; Morsci et al., 2016), revealing that mitochondria form discrete puncta in axonal processes and more complex networks in cell bodies. We confirmed these observations using a pan-neuronally expressed mitochondrial reporter P*rgef-1*-mito-GFP(*juEx7517*) (Figure S1A) as well as a touch neuron specific marker P*mec-4*-mito-GFP(*juEx3328*) (Figure S1B). The density of mitochondrial puncta in the proximal PLM axon was approximately one per ten microns (Figure S1C), which is similar to that recently reported for *C. elegans* motor neurons (Han et al., 2016). Mitochondrial density in the distal axon of ALM neurons has been reported to increase during adult life (Morsci et al., 2016), yet we observed that the mitochondrial density in proximal PLM axons remains stable through the seventh day of adulthood (Figure S1C), suggesting that mitochondrial density may be differentially regulated between different neuron types, or even between distal and proximal parts of the same axon.

**Figure 1:**
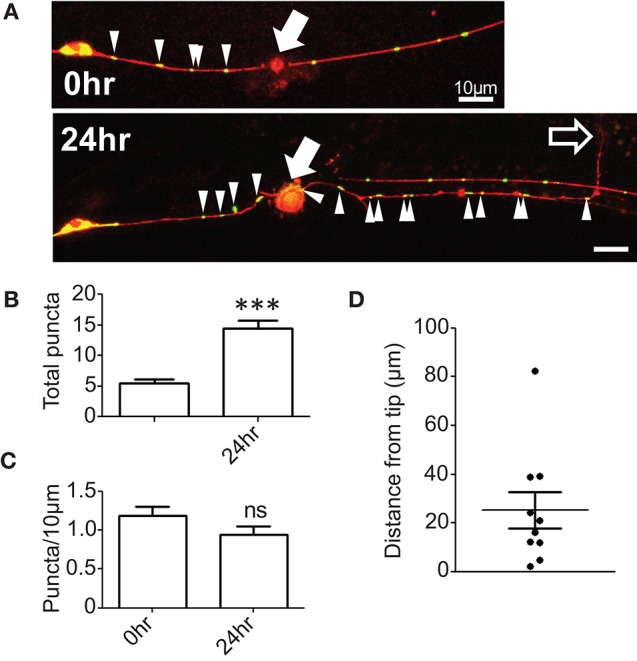
**Mitochondrial density is maintained in regenerating axons. (A)** PLM axons were visualized with P*mec-4*-TagRFP(*juIs252*) and mitochondria with P*mec-4*-mitoGFP(*juEx3328*). Axons were severed ~50 μm from the cell body and the number of mitoGFP puncta and the length of the regrowing axon quantified 24 h post injury. **(B)** Quantification of total number of mitoGFP puncta in the portion of axon attached to the cell body (proximal axon) 24 h post injury. **(C)** Mitochondrial density was calculated as the total number of puncta divided by the length of the entire proximal axon, normalized to 10 μm. **(D)** Quantification of the distance between the regrowing axon tip or growth cone and the closest mitochondrial punctum. *n* = 10 animals tested, *p*-value from paired *t*-test: ns, not significant, ^***^p < 0.001. Filled arrows mark injury site, arrowheads indicate mitoGFP puncta in proximal axon, and unfilled arrow indicates tip of regrowing axon. Scale bars = 10 μm.

To examine whether mitochondrial distribution changes in response to axon injury we performed laser axotomy at the fourth larval stage, immediately before the final molt to adulthood. We observed an increase in the number of mitochondrial puncta in the proximal axon 24 h after injury (Figures 1A,B); however, this increase was proportional to axon regrowth such that the density of axonal mitochondria in the regrowing axon was maintained at pre-injury levels (Figure 1C). These observations suggest that the regrowing PLM axon maintains a constant mitochondrial density during regrowth, possibly by either increasing mitochondrial biogenesis or transport from the cell soma. Additionally, we noticed that mitochondria were generally not located at the regrowing tips of axons, but rather were variable distances away from the growth cone (Figures 1B,D). This is in contrast to a recent report wherein regrowing commissural axons of *C. elegans* motor neurons exhibit a marked increase in the density of mitochondrial puncta after injury as a result of increased cellular transport as well as localization of mitochondria to the growth cone (Han et al., 2016). These two studies in different neural subsets suggest that the density and localization of mitochondria after injury may be controlled in a cell type-specific manner. Regardless, the maintenance or increase of axonal mitochondrial density after injury (as opposed to decrease) in PLM neurons suggests that these organelles are important in axon regrowth. We therefore took a genetic approach to define which aspects of mitochondrial function might be relevant in axon regeneration.

### A survey of mitochondria-related genes: transport, calcium uptake, and biogenesis

To learn how mitochondria contribute to axon regrowth, we surveyed genes whose orthologs are known to affect mitochondrial biogenesis, transport, or calcium uptake (Table 1). In addition, we performed a more extensive survey of genes involved in the electron transport chain (see next section; Table 2). As in our previous large-scale mutant screen (Chen et al., 2011), our analysis was confined to viable mutants; Because mitochondrial function is essential for viability (Tsang and Lemire, 2003), we exploited null mutants in non-essential genes or partial loss of function mutants in those genes that are essential to animal development and survival.

**Table 1:** **Mitochondrial pathways screened for axon regeneration**.

**Gene (allele)**	**Human ortholog**	**Pathway**	**Regrowth (% of WT)**	***n***	***p*-value**
*miro-1(tm1966)*	*RHOT1*	Transport	114.8 ± 7.8	22	0.1587
*miro-2(tm2933)*	*RHOT2*	Transport	110.7 ± 7.9	26	0.2907
*trak-1(tm1572)*	*TRAK1*	Transport	104.4 ± 3.3	22	0.7081
*mcu-1(ju1154)*	*MCU*	Calcium	82.96 ± 3.2	22	0.1174
*emre-1(tm6230)*	*SMDT1*	Calcium	92.09 ± 7.4	29	0.3975
*pink-1(tm1779)*	*PINK1*	Mitophagy	72.65 ± 5.8	26	^**^
*pink-1(ok3538)*	*PINK1*	Mitophagy	88.42 ± 3.8	28	0.2247
*pdr-1(gk448)*	*PARK2*	Mitophagy	110.7 ± 2.9	24	0.2895
*pdr-1(tm395)*	*PARK2*	Mitophagy	96.68 ± 2.9	23	0.7505
*pdr-1(tm598)*	*PARK2*	Mitophagy	95.15 ± 4.0	20	0.6758
*drp-1(tm1108)*	*DNM1L*	Fission	94.1 ± 4.0	23	0.3419
*fis-1(tm1867)*	*FIS1*	Fission	113. ± 6.6	22	0.1775
*fis-1(tm2227)*	*FIS1*	Fission	108.8 ± 7.3	21	0.4543
*fis-2(gk363)*	*FIS1*	Fission	107.9 ± 8.3	22	0.4745
*fis-2(gk414)*	*FIS1*	Fission	115.0 ± 6.4	41	0.0735
*fis-2(tm1832)*	*FIS1*	Fission	107.5 ± 8.5	28	0.4462
*fzo-1(tm1133)*	*MFN2*	Fusion	87.33 ± 5.4	19	0.074
*eat-3(ad426)*	*OPA1*	Fusion	62.02 ± 6.2	17	^***^
*eat-3(tm1107)*	*OPA1*	Fusion	70.27 ± 4.2	26	^***^

Human orthologs of C. elegans genes, as well as their mitochondrial pathway are listed. Regrowth is reported as mean percentage of wild type (WT) ± S.E.M., n = number of animals tested, and p-value from Student's t-test of mutant vs. same-day control,

** p < 0.01,

*** *p < 0.001*.

**Table 2:** **Mitochondrial electron transport chain genes tested**.

**Gene (allele)**	**Human ortholog**	**ETC complex**	**Regrowth (% of WT)**	***n***	***p*-value**
*gas-1(fc21)*	*NDUFS2*	I	70.0 ± 6.0	25	^***^
*nduf-2.2(ok437)*	*NDUFS2*	I	41.7 ± 5.3	25	^***^
*nduf-7(et19)*	*NDUFS7*	I	67.5 ± 3.8	23	^***^
*nuo-6(qm200)*	*NDUFB4*	I	106.1 ± 6.2	24	0.4557
*mev-1(kn1)*	*SDHC*	II	94.0 ± 7.5	21	0.5693
*sdha-2(tm1420)*	*SDHA*	II	97.6 ± 6.0	22	0.8019
*clk-1(e2519)*	*COQ7*	Ubiquinone	91.1 ± 4.7	24	0.2637
*clk-1(qm30)*	*COQ7*	Ubiquinone	91.3 ± 4.5	29	0.212
*rad-8(mn163)*	*RTN4IP1*	II/III	51.5 ± 7.4	25	^***^
*isp-1(qm150)*	*UQCRFS1*	III	23.3 ± 3.9	27	^***^
*ucr-2.3(ok3073)*	*UQCRC2*	III	106.5 ± 5.9	30	0.4429
*ucr-2.3(pk732)*	*UQCRC2*	III	83.05 ± 5.5	27	0.0656
*asg-2(ok3344)*	*ATP5L*	ATP synthase	82.84 ± 5.6	26	0.0908
*asg-2(tm1472)*	*ATP5L*	ATP synthase	82.5 ± 6.2	21	0.1068

Human orthologs of C. elegans electron transport chain genes and their complex association are listed. Regrowth is reported as mean percentage of wild type (WT) ± S.E.M., n = number of animals tested, and p-value from Student's t-test of mutant vs. same-day control,

*** *p < 0.001*.

We first focused on genes implicated in microtubule-dependent transport of mitochondria. The Miro and Trak/Milton protein families act as adaptors between mitochondria and microtubule-binding molecular motors and are required in other organisms for anterograde and retrograde transport of mitochondria in axons (Schwarz, 2013). The *C. elegans* genome contains two Miro family members *miro-1* and *miro-2* (Shen et al., 2016; Xu et al., 2016) and a single Trak/Milton family member *trak-1* (Mercer et al., 2009). *miro-1(tm1966)* null mutants appear superficially wild type, but have been shown to have increased longevity and reduced mitochondrial content (Shen et al., 2016), as well as altered mitochondrial morphology in the epidermis (Xu et al., 2016). *miro-2(tm2933)* null mutants are superficially wild type and have normal epidermal mitochondrial morphology (Xu et al., 2016). Single mutants in *miro-1* or *miro-2* displayed normal PLM axon regeneration, suggesting individual Miro genes are not required for regrowth. *trak-1(tm1572)* null mutants also displayed normal PLM axon regeneration. Together these data suggest that despite the observed increase in the number of mitochondrial puncta in the regrowing axons, and in contrast to the recently reported role for *miro-1* in motor axon regeneration (Han et al., 2016), individual transport adaptors are dispensable for PLM axon regeneration.

One of the initial signals of axonal damage is an increase of axonal calcium that spreads wave-like bi-directionally away from the site of injury (Ghosh-Roy et al., 2010). Injury-triggered calcium transients are also observed in *C. elegans* epidermal wound responses, where they trigger local mitochondrial calcium uptake and reactive oxygen species (ROS) production critical for wound repair (Xu and Chisholm, 2014). To test whether mitochondrial calcium pathways might be involved in axon repair or regeneration, we tested null mutations in the mitochondrial calcium uniporter (MCU) ortholog *mcu-1* and in the essential MCU regulator (EMRE) ortholog *emre-1*, required for coupling uniporter opening to calcium-sensing subunits (Sancak et al., 2013). These *mcu-1* and *emre-1* mutants displayed normal PLM axon regrowth, consistent with observations in *mcu-1* mutants in motor axon regrowth (Han et al., 2016), suggesting that mitochondrial calcium uptake is not a critical determinant of axon regeneration.

We next tested *C. elegans* homologs of genes involved in mitophagy, the breakdown of faulty mitochondria. In mammals and *Drosophila* the PINK1 serine/threonine kinase activates the ubiquitin-ligase activity of PARKIN/PDR-1, which marks mitochondria for degradation (Song et al., 2013). The *C. elegans* PINK1 ortholog *pink-1* and the Parkin/PDR1 ortholog *pdr-1* have been implicated in mitophagy and mitochondrial biogenesis (Palikaras et al., 2015; Pickrell and Youle, 2015). We tested two *pink-1* deletions, both of which are presumed null mutants: *pink-1(tm1779)* (Samann et al., 2009) displayed significantly reduced axon regeneration, yet *pink-1(ok3538)* (Valenci et al., 2015) displayed normal axon regrowth. We note that the *tm1779* deletion also affects the inter-genic region of the operon that includes the 3′ untranslated region of the upstream F-box gene EEED8.10; *tm1779* may affect expression of the downstream gene(s), or *tm1779* strains may contain background mutations that we were unable to eliminate in outcrossing. Axon regrowth was normal in three independent alleles of the *PARKIN* homolog *pdr-1*, all of which are thought to cause strong loss of function at the protein level (Springer et al., 2005; Valenci et al., 2015). Together, we conclude that mitophagy-related genes are not required for axon regeneration.

Finally, we tested *C. elegans* genes implicated in mitochondrial fission and fusion (Mishra and Chan, 2014), namely *drp-1/DNM1L* and the *FIS1* homologs *fis-1* and *fis-2* for fission, and *fzo-1/MFN1* and *eat-3/OPA1* for fusion (Table 1). While *drp-1* null mutants have an abnormally fused mitochondrial network, the network in *fis-1* or *fis-2* null mutants is normal; FIS-1 and FIS-2 have been suggested to play a role in mitophagy-related mitochondrial fission rather than mitochondrial network maintenance (Breckenridge et al., 2008; Shen et al., 2014). When tested for axon regrowth after injury, all these fission-defective mutants displayed normal regrowth phenotypes (Table 1). We next examined a null allele of *fzo-1* and two loss-of-function alleles of *eat-3/Opa1*, orthologs of which mediate fusion of the outer and inner mitochondrial membranes, respectively (Breckenridge et al., 2008; Kanazawa et al., 2008; Rolland et al., 2009). Interestingly, *eat-3*, but not *fzo-1*, mutants displayed significantly reduced axon regrowth. As *fzo-1* and *eat-3* mutants exhibit comparably fragmented mitochondrial networks in other cell types (Breckenridge et al., 2008), the requirement for *eat-3* in regrowth may be independent of its role in mitochondrial fusion. The *eat-3* ortholog Opa1 functions in remodeling the cristae of the inner mitochondrial membrane, independent of its role in IMM fusion (Cogliati et al., 2013; Pernas and Scorrano, 2016). Since cristae house the electron transport chain, and our previous and current screen implicated ETC function in axon regrowth, we hypothesized that the requirement for *eat-3* in axon regrowth is indirect, via its effects on the ETC. However, mutants of *immt-1, immt-2, moma-1*, and *chch*-3, genes which have been implicated in cristae formation or maintenance (Mun et al., 2010; Head et al., 2011), all showed normal axon regrowth (Figure S2). Nevertheless, and in light of the two genes identified in the original screen belonging to the ETC, we next turned our attention to ETC genes and whether ETC function is critical for axon regrowth.

### Select components of the electron transport chain are required for axon regeneration

**Figure 2:**
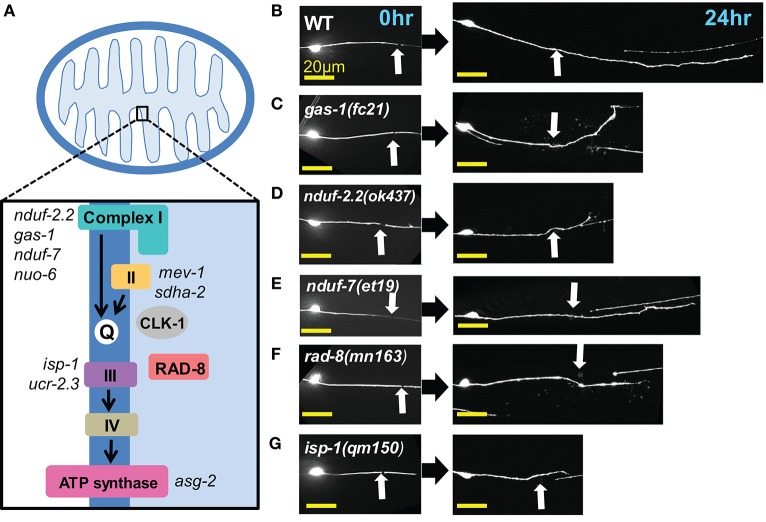
**Axon regeneration is reduced in a subset of electron transport chain gene mutants. (A)** Diagram of the electron transport chain located on the inner mitochondrial membrane, with ETC genes tested indicated next to their complex. **(B–G)** Representative images of wild type (WT) and the five ETC mutants with significantly decreased axon regeneration 24 h after injury. Arrow indicates injury site. Scale bars = 20 μm.

Four multiprotein complexes (Complexes I–IV) work to metabolize products from the citric acid cycle in order to create an electrochemical gradient across the inner mitochondrial membrane, which in turn powers the ATP synthase (Complex V) that generates ATP to power cellular reactions (Figure 2A). While complete loss of function in mitochondrial electron transport is lethal (Tsang and Lemire, 2003), viable mutations in a number of *C. elegans* ETC components have been identified, many of which have been studied for their effects on animal lifespan (Munkacsy and Rea, 2014; Dancy et al., 2015). Besides the previously tested genes *nduf-2.2* and *isp-1*, we tested mutants in nine additional ETC components, some of which have been shown biochemically by others to display reduced ETC function (Table 2). Of the new mutants tested, only three had significantly reduced axon regrowth: the Complex I iron-sulfur protein-encoding genes *gas-1* and *nduf-7* and the putative mitochondrial oxidoreductase *rad-8* (Figures 2B–G).

**Figure 3:**
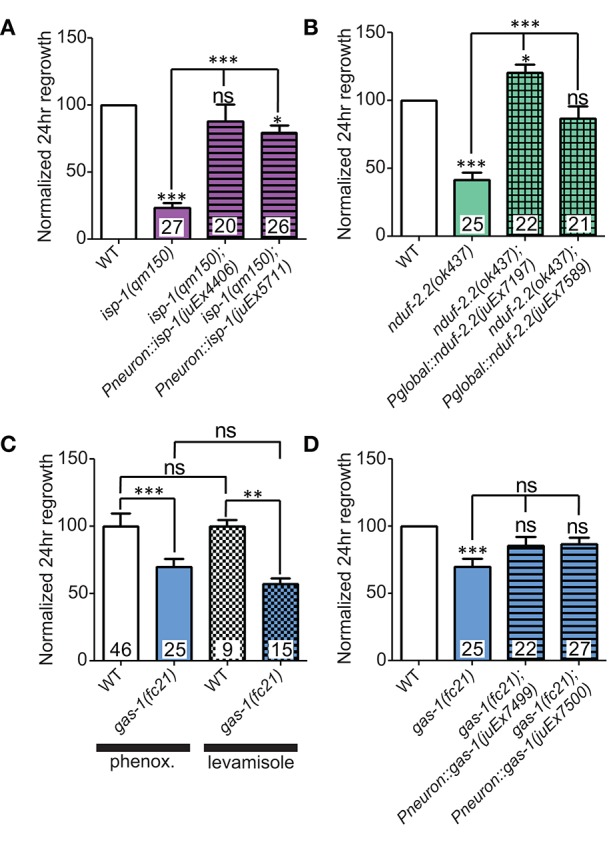
**Iron-sulfur proteins can be rescued with genomic sequences**. Two independently-generated pan-neuronal or global transgenic arrays of wild type genomic sequences were able to rescue the mutants **(A)** *isp-1(qm150)* and **(B)** *nduf-2.2(ok437)*, respectively. **(C)** Testing *gas-1(fc21)* mutants using either 0.1% phenoxypropanol or 0.1% levamisole revealed that the impairments in regeneration in this mutant are independent of the immobilization method used. **(D)** Pan-neuronal transgenic arrays containing the wild type genomic sequence of *gas*-1 were able to partially restore axon regrowth in *gas-1(fc21)* mutants. Lower tier *p*-values calculated by Student's *t*-test vs. same-day control and upper tier *p*-values calculated by *ANOVA* with Tukey's multiple comparison post-test across mutant strains: ns, not significant, ^*^p < 0.05, ^**^p < 0.01 ^***^p < 0.001.

We first extended our previous observations of impaired regrowth in *isp-1* and *nduf-2.2* mutants. The *isp-1(qm150)* allele is a point mutation that reduces Complex III function and lowers overall cellular respiration by 40% (Feng et al., 2001). Axon regrowth defects seen in this mutant were completely rescued by expression of wild type *isp-1* under the control of a pan-neuronal promoter (Figure 3A), suggesting a cell-autonomous requirement for ETC function in axon regrowth.

*C. elegans* encodes two orthologs of the NDUFS2 subunit of complex I: *nduf-2.2* and *gas-1* (Kayser et al., 2001). *gas-1* is ubiquitously expressed, whereas the expression pattern of *nduf-2.2* is undetermined (Kayser et al., 2004). The *nduf-2.2(ok437)* allele is a large deletion and likely null with unknown effects on mitochondrial metabolism, although mutants appear superficially wild type. In contrast, *gas-1(fc21)* partial loss of function mutants are small and slow-growing and display drastically reduced Complex I activity (Kayser et al., 1999, 2001). Consistent with previous observations, *nduf-2.2(ok437)* reduced regrowth by roughly 60%, and expression of wild type *nduf-2.2* under the control of a ubiquitously expressed promoter fully rescued axon regrowth phenotypes (Figure 3B). *gas-1(fc21)* mutants, which grow slowly and have a small body phenotype, also displayed a defect in PLM axon regrowth (Table 2), although not as severe as in *nduf-2.2* mutants. This suggests that defective axon regrowth is not the result of organismal growth rate and that *nduf-2.2* may play the predominant role in regrowing axons. As *gas-1(fc21)* was originally isolated based on hypersensitivity to anesthetics (Kayser et al., 1999), we tested its effects on axon regrowth using two independent immobilization agents, phenoxypropanol and levamisole, with comparable results (Figure 3C). Pan-neuronal expression of wild type *gas-1* was sufficient to rescue *gas-1(fc21)* axon regrowth defects to normal levels (Figure 3D). A recent report identified the *et19* partial loss-of-function allele of *nduf-7*, another Complex I iron-sulfur protein (Rauthan et al., 2015). *nduf-7(et19)* mutants are viable and healthy yet slightly slow-growing, and displayed defective axon regrowth (Figure 2E). Together, these data suggest the ETC Complex I iron-sulfur protein subunits are important for axon regeneration.

The above results on subunits of the ETC Complexes I and III are consistent with a general requirement for mitochondrial respiration and ATP synthesis in axon regrowth. Indeed, recent results showing a requirement for mitochondria in motor axon regrowth have been interpreted as reflecting the high energetic requirements in this process (Han et al., 2016). To further address the role of the ETC in axon regrowth we examined additional ETC components (Table 2). Unexpectedly, loss of function in most of these genes had no effect on PLM axon regeneration: In Complex I, we tested *nuo-6/NDUFB4*(*qm200)*, which exhibits reduced Complex I activity and overall mitochondrial respiration, as well as impaired motor axon regrowth after injury (Yang and Hekimi, 2010b; Han et al., 2016). In Complex II we tested the *kn1* allele of *mev-1*/*SDHC*, which displays decreased Complex II function but normal ATP levels (Ishii et al., 1998). Also in Complex II, the *tm1420* allele of *sdha-2*/*SDHA*, has a 519 bp in-frame deletion in exon four, which removes most of the FAD-binding domain of the protein and thus likely rendering it unable to oxidize succinate. We tested two alleles of the Complex III core protein *ucr-2.3*/*UQCRC2*l: the *ok3073* allele is a 415 bp deletion and 4 bp insertion creating a premature stop codon; *pk732* is a point mutation in the insulinase domain (Butler et al., 2010). No viable alleles in genes encoding subunits of Complex IV were available. For the ATP synthase, we tested two deletion alleles of the γ-subunit homolog *asg-2/ATP5L*. We also tested *clk-1/COQ7*, which catalyzes the final step in the synthesis of ubiquinone, an essential electron carrier that shuttles electrons from Complexes I or II to Complex III (Felkai et al., 1999). *clk-1(e2519)* is a point mutation in the active site and is defective in conversion of 5-demethoxyubiquinone into ubiquinone, while the *clk-1(qm30)* deletion is a null (Ewbank et al., 1997; Miyadera et al., 2001; Branicky et al., 2006). Collectively, these mutants have varying effects on ETC function and ATP production (Dancy et al., 2015), yet all had largely normal axon regeneration 24 h after axotomy, contradicting our hypothesis that the ETC is required for axon regeneration (Table 2).

**Figure 4:**
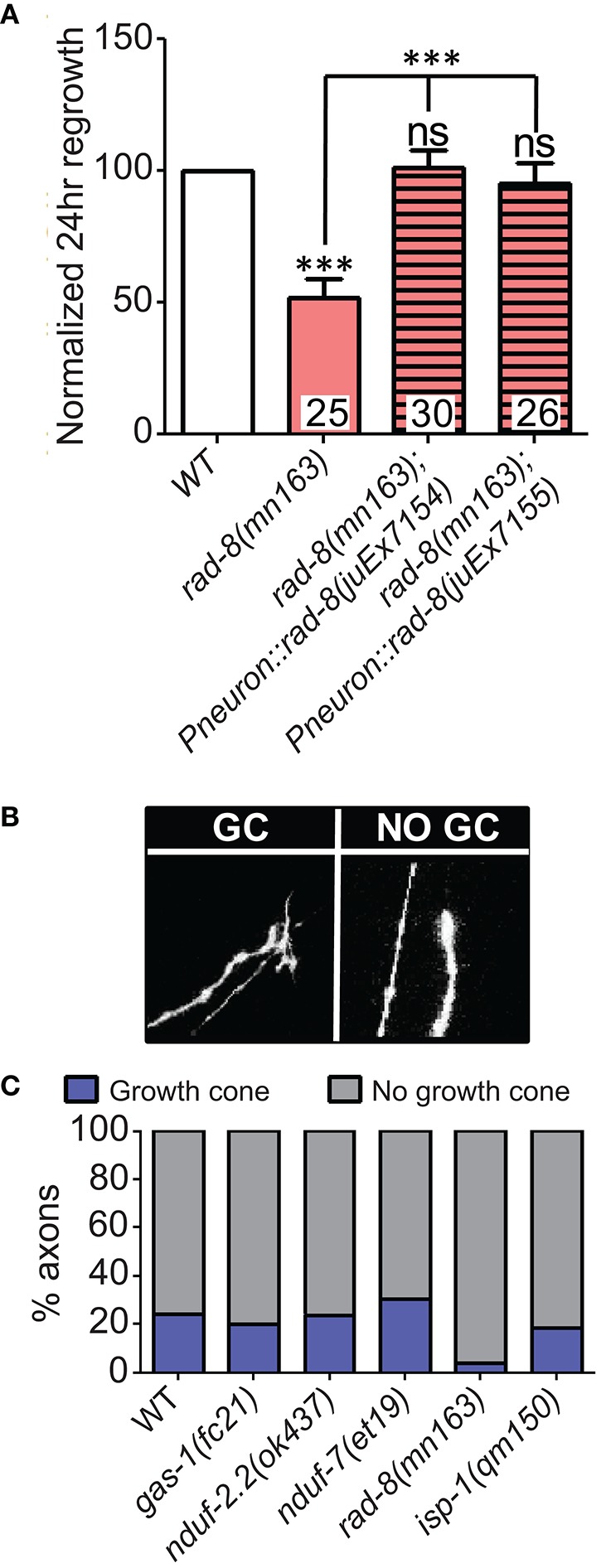
***rad*****-8 mutants can be rescued with genomic sequence and exhibit fewer growth cones. (A)** Wild type genomic sequence of *rad-8* expressed pan-neuronally can rescue *rad-8(mn163)* axon regeneration to normal levels. **(B)** Examples of regrowing axon terminals with (left) or without (right) growth cones. **(C)** Proportions of ETC mutant animals with axons terminating with (blue) or without (gray) growth cones at 24 h post-injury, with only *rad-8* mutants showing fewer growth cones than wild type. Lower tier *p*-values calculated by Student's *t*-test vs. same-day control and upper tier *p*-values calculated by *ANOVA* with Tukey's multiple comparison post-test across mutant strains: ns, not significant, ^***^p < 0.001.

Among the additional ETC components tested, only *rad-8* mutants displayed defective regrowth (Figure 2F, Table 2). *rad*-*8* encodes a putative mitochondrial oxidoreductase, and the *mn163* mutation results in a premature stop codon and is a presumed null allele. This mutant has decreased electron transfer from Complex II to Complex III (Fujii et al., 2011), but normal ATP levels (Braeckman et al., 2000); the precise role of RAD-8 in electron transport is unclear. Pan-neuronal expression of wild type *rad-8* was sufficient to rescue *rad-8* axon regrowth defects (Figure 4A). Unlike *isp-1* and other ETC mutants, regrowing PLM axons in *rad-8(mn163)* displayed a reduced frequency of growth cones 24 h after injury (4 vs. 20% in wild type; Figures 4B,C).

Overall, since many of the ETC mutants have normal axon regrowth, our data suggest that PLM axon regeneration is not simply dependent on ATP generation by the entire ETC, but rather on the function of specific subset of ETC components. The relationship between dysfunction of a small group of ETC components and overall mitochondrial metabolism is complex, thus it may be that these genes have a special function within the ETC, or that their loss or mutation leads to cellular adaptations which inhibit axon regrowth (see below).

### Mitochondrial ETC genes are required for axon extension and act downstream or in parallel to injury signals

**Figure 5:**
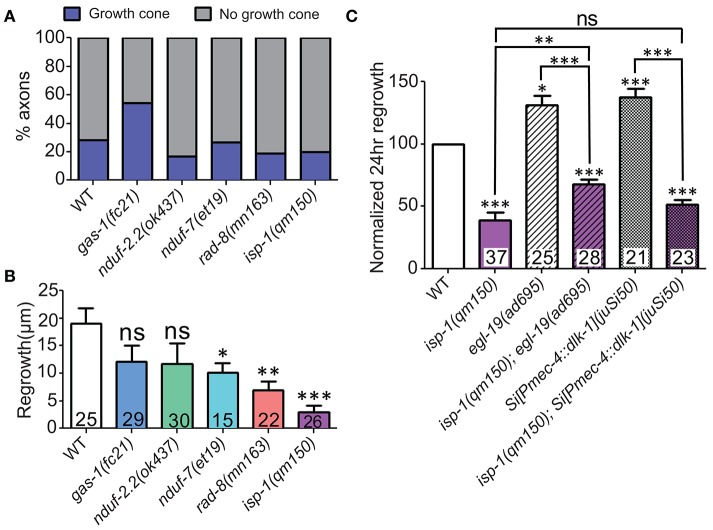
**ETC genes affect axon extension, downstream of early injury signaling. (A)** The proportions of ETC mutant animals with axons terminating with (blue) or without (gray) growth cones at 6 h post-injury are largely normal, suggesting that all mutants can respond to injury by forming growth structures. **(B)** Measuring axon length at 6 h post-injury reveals deficits in the ETC mutants, indicating defects in axon extension. **(C)** The *isp-1(qm150)* mutation can partially or fully block the enhanced regeneration phenotypes of either *egl-19(gf)* or two extra copies of *dlk-1* in mechanosensory neurons, respectively. Colored bars in **(C)** indicate presence of *qm150* mutant allele. Lower tier *p*-values calculated by Student's *t*-test vs. same-day control and upper tier *p*-values calculated by *ANOVA* with Tukey's multiple comparison post-test across mutant strains: ns, not significant, ^*^p < 0.05, ^**^p < 0.01, ^***^p < 0.001.

To get a deeper understanding of the regeneration defects in the affected ETC mutants, we next asked whether mitochondria are required for early responses such as formation of growth cones at the tip of the regrowing axon. Defective axon regrowth 24 h after injury could result from the failure of a number of steps in regrowth, from initial injury detection and signal propagation to growth cone formation and axon extension. These five ETC mutants with defective axon regrowth had a normal frequency of growth cone formation at 6 h after injury (Figure 5A). Furthermore, by this time point, regrowing wild type axons had extended nearly 20 μm, whereas the ETC mutants consistently displayed reduced axon extension (Figure 5B). Altogether these data suggest that the ETC genes are not required in injury detection and growth cone formation, rather they likely function in the extension phase of axon regeneration.

Elevated calcium influx after injury with a gain-of-function mutation in the voltage gated calcium channel EGL-19 or elevated injury signaling by overexpression of *dlk-1* each enhance axon regrowth beyond wild type levels (Ghosh-Roy et al., 2010; Yan and Jin, 2012). To test whether the ETC functions downstream of the initial injury signaling, we performed double mutant analyses between *isp-1(qm150)* mutants and either *egl-19(gf)* or DLK-1 overexpressing animals. Double mutants of *isp-1(qm150)* and the *egl-19(ad695)* mutant were strongly defective in regrowth, although not as strongly as *isp-1(qm150)* alone (Figure 5C). Similarly, *isp-1(qm150)* was almost fully epistatic to the effects of *dlk-1* overexpression. We conclude that the role of mitochondria in axon regeneration lies downstream of initial injury signals and that boosting these early events cannot bypass the requirement for mitochondria in later axon extension. The partial epistasis of *isp-1* with *egl-19(gf)* is consistent with multiple pathways acting downstream of injury signals.

### Overexpression of *nduf-2.2* or *isp-1* can enhance axon regeneration

**Figure 6:**
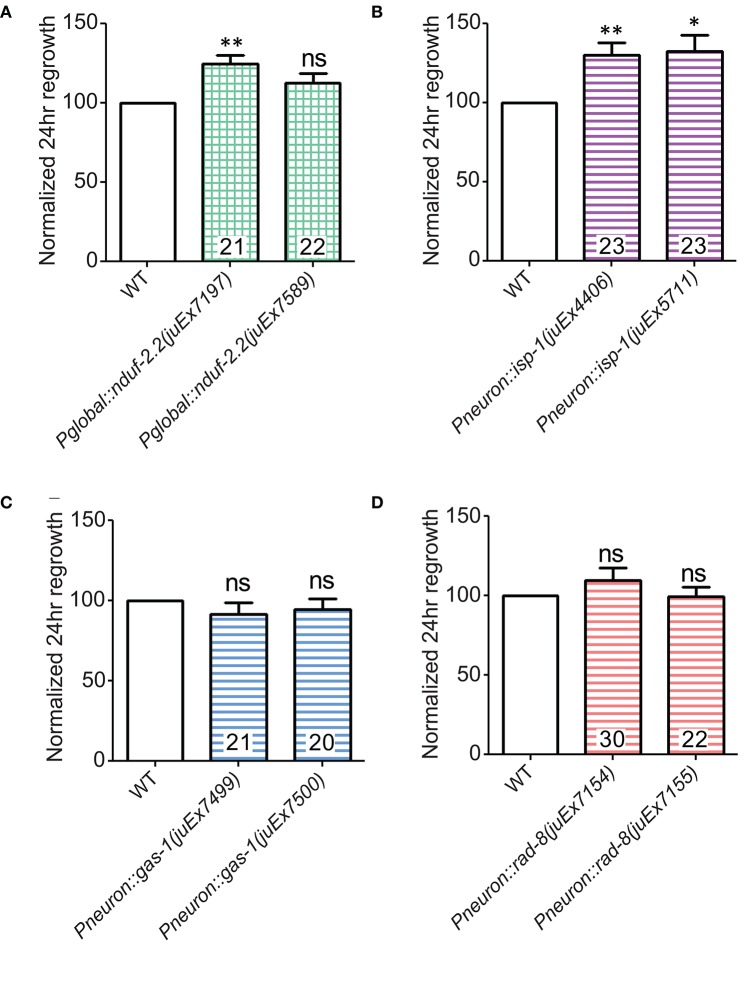
**Overexpression of either *nduf-2.2* or *isp-1* enhances regeneration in the wild type background. (A)** One of the mutant-rescuing arrays of *nduf-2.2* under a global promoter enhanced axon regrowth in the wild type background. **(B)** Both transgenic arrays with pan-neuronal overexpression of *isp-1* were sufficient to increase regrowth in the wild type background. However, neuronal overexpression of neither **(C)** *gas-1* nor **(D)** *rad-8* had a significant effect in wild type animals. *p*-values were calculated using Student's *t*-test vs. same-day control: ns, not significant, ^*^p < 0.05 ^**^p < 0.01.

During the above rescue experiments, we noticed that animals expressing one of the *nduf-2.2* transgenic arrays displayed axon regrowth significantly higher than wild type, even in the *nduf-2.2(ok437)* background (Figure 3B). We explored whether overexpressing these ETC genes in the wild type background would affect axon regeneration. Interestingly, rescuing transgenes for the iron-sulfur proteins *nduf-2.2* and *isp-1* using either a ubiquitous or a pan-neuronal promoter, respectively, enhanced PLM axon regeneration in the wild type background (Figures 6A,B). Although, *nduf-2.2* and *gas-1* are over 90% identical in amino acid sequence and share similar functions (Kayser et al., 2001), transgenes of wild type *gas-1* using a pan-neuronal promoter did not enhance axon regeneration in the wild type background (Figure 6C). Similarly, neuronal overexpression of wild type *rad*-*8* also had no effect (Figure 6D). With the caveat that expression levels have not been directly measured in these strains, our data suggest that overexpression of *nduf-2.2* or *isp-1* can be sufficient to enhance axon regrowth above normal levels.

### Interactions of *rad-8* with the mitochondrial electron transport chain

Our finding that *rad-8* is required for efficient axon regrowth prompted us to investigate this gene in more depth, since the cellular function of RAD-8 remains unclear. *rad-8* mutants were originally isolated by virtue of their hypersensitivity to radiation (Hartman and Herman, 1982) and later found to exhibit reduced electron transport between Complexes II and III (Fujii et al., 2011). *rad-8* encodes a putative mitochondrial dehydrogenase/reductase (Fujii et al., 2011) related to the mammalian Nogo-interacting protein RTN4IP/NIMP (Hu et al., 2002). To assess whether RAD-8 is required in mitochondria, we mis-targeted the protein to the cytoplasm by deleting the N-terminal mitochondrial localization sequence (MLS) as described by Hu et al. (2002). Wild type animals expressing RAD-8ΔMLS pan-neuronally displayed normal development and behavior, but in the *rad-8(mn163*) background the RAD-8ΔMLS transgene significantly decreased viability, precluding axon regeneration testing. These experiments suggest the N-terminal MLS is important for RAD-8 function, consistent with a role in mitochondria.

**Figure 7:**
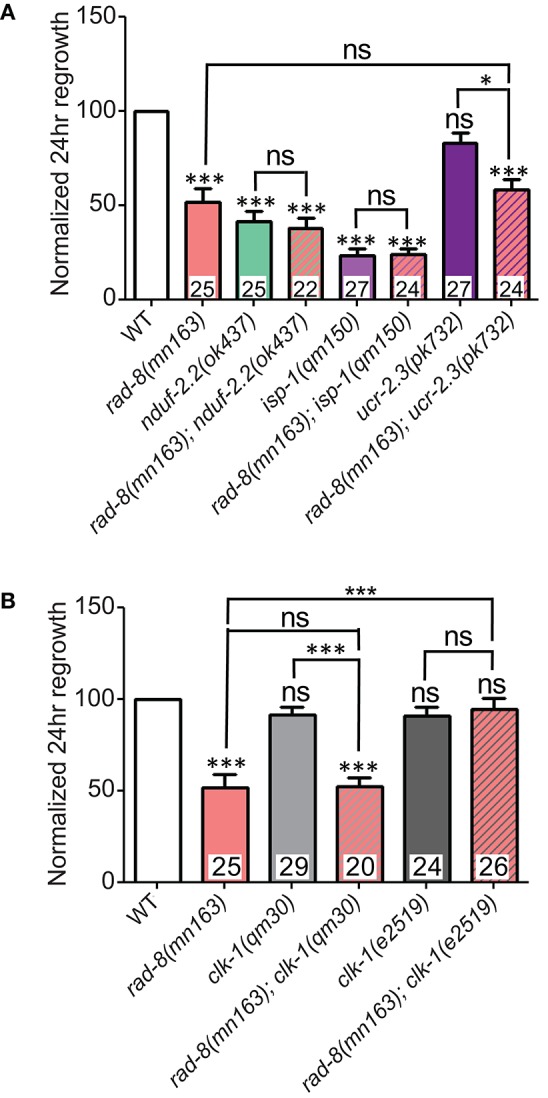
***rad-8*** **epistasis places it in the same pathway as other ETC genes. (A)** Axon regrowth in double mutants of *rad-8(mn163)* with either *nduf-2.2(ok437)* or *isp-1(qm150)* were identical to either *nduf-2.2(ok437)* or *isp-1(qm150)* single mutants, while double mutants of *rad-8(mn163)* with *ucr-2.3(pk732)* were identical to *rad-8(mn163)* single mutants. **(B)** *rad-8(mn163); clk-1(qm30)* double mutants were identical to *rad-8(mn163)* single mutants, while *rad-8(mn163); clk-1(e2519)* double mutants had wild type levels of axon regrowth. Lower tier *p*-values calculated by Student's *t*-test vs. same-day control and upper tier *p*-values calculated by *ANOVA* with Tukey's multiple comparison post-test across mutant strains: ns, not significant, ^*^p < 0.05, ^***^p < 0.001.

We next constructed compound mutants of *rad-8(mn163)* with other ETC mutants. Double mutants between *rad-8(mn163)* and *nduf-2.2(ok437)* or *isp-1(qm150)* were extremely slow growing yet displayed sub-additive interactions in axon regrowth, such that the double mutants resembled the strongest single mutant phenotype (Figure 7A). We were unsuccessful in our attempts to generate viable double mutants between *rad-8(mn163)* and *gas-1(fc21), mev-1(kn1)*, and the C-terminal deletion mutant *ucr-2.3(ok3073)*. Double mutants of *rad-8(mn163)* with *ucr-2.3(pk732)*, a point mutant in the insulinase domain of a Complex III core subunit that is phenotypically similar to *mev-1(kn1)* (Butler et al., 2010) and had normal axon regrowth, showed axon regeneration levels similar to *rad-8(mn163)* mutants.

Additionally, we tested whether *rad-8(mn163)* interacts with *clk-1*. As noted above, the null allele *clk-1(qm30)* had no effect on axon regeneration alone, and double mutants resembled *rad-8(mn163)* in regrowth (Figure 7B). Unexpectedly, when *rad-8(mn163)* was combined with the *clk-1(e2519)* point mutation, the *rad-8(mn163)* axon regeneration defect was suppressed to wild type levels. Both *clk-1* alleles partially suppressed the slow growth and small body size of the *rad-8(mn163)* mutants when grown at 20°C, but only *e2519* suppressed *rad-8* axon regrowth defects, further supporting a conclusion that axon regrowth is separable from organismal growth rate.

### Inhibiting the mitoUPR suppresses *nduf-2.2* regeneration defects

**Figure 8:**
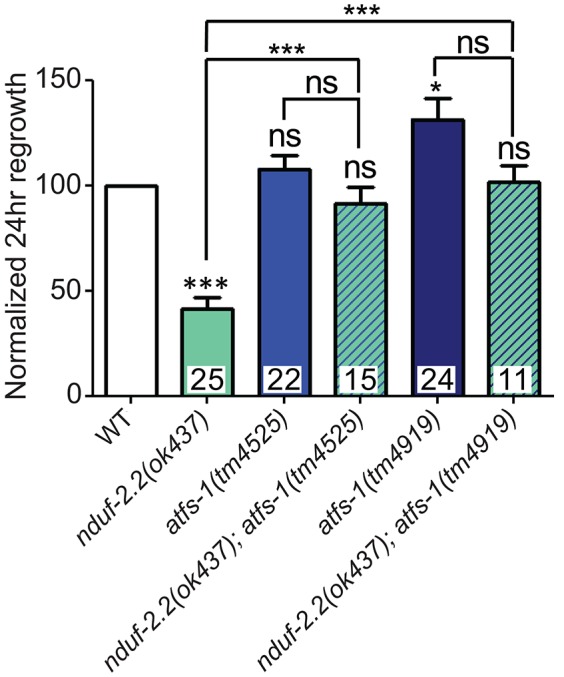
**Mutations in the mitoUPR response gene *atfs-1* suppress axon regeneration defects in *nduf-2.2*.** Two deletion mutants of *atfs-1* have either no effect or a small increase in axon regeneration on their own, but double mutants of either *atfs-1* allele and *nduf-2.2* have wild type axon regrowth. Lower tier *p*-values calculated by Student's *t*-test vs. same-day control and upper tier *p*-values calculated by *ANOVA* with Tukey's multiple comparison post-test across mutant strains: ns, not significant, ^*^p < 0.05, ^***^p < 0.001.

One response to mitochondrial dysfunction is the mitochondrial unfolded protein response (mitoUPR), in which the transcription factor ATFS-1 is released from mitochonrdria and translocates to the cell nucleus to turn on the expression of adaptive genes (Nargund et al., 2012; Kornmann, 2014). We tested two deletion alleles of *atfs-1* that affect the N-terminal region of the gene, likely causing strong loss of function. We observed a mild improvement in axon regeneration in one allele, and another allele showed wild type levels of regrowth (Figure 8), suggesting that inhibiting the mitoUPR has no major effect on axon regeneration. However, double mutants of either *atfs-1* allele with the ETC gene *nduf-2.2* showed suppression of the regeneration defect seen in *nduf-2.2* single mutants. Our attempts to generate double mutant strains between *atfs-1* and the other affected ETC genes were unsuccessful because these mutants were either extremely slow-growing or lethal. Together with the interaction between *rad-8* and *clk-1* observed above, these data suggest that absence of *rad-8* or *nduf-2.2* likely trigger special cellular adaptations and stress signaling cascades, which are compensated upon impairment in mitoUPR function, resulting in normal regrowth of injured axons.

## Discussion

Increasing evidence from diverse model systems points to an important role for mitochondria in regenerative axon regrowth. Here, building on initial observations from our large-scale axon regeneration screen (Chen et al., 2011), we explored the role of mitochondria in depth. Using *C. elegans* mechanosensory neurons, we find that injured neurons maintain their axonal mitochondrial density as they regrow and do not send mitochondria to the tips of regrowing axons. Regeneration is generally resistant to loss-of-function mutations in most mitochondrial biogenesis or fission/fusion pathways, with the exception of *eat-3*/Opa1, which may affect assembly of the electron transport chain. We identify a subset of ETC components required for efficient regrowth, and show that these likely act cell autonomously during the axon extension phase of regrowth.

We find that in *C. elegans* PLM mechanosensory neurons, axon injury does not trigger dramatic alterations in mitochondrial distribution. By 24 h post injury, the total number of axonal mitochondria in the regrowing axon has increased so as to maintain mitochondrial density at ~10 per 100 μm of axon length, with the most distal mitochondrion located ~25 μm from the regrowing axon tip. These observations may be compared with recent studies of *C. elegans* motor neuron commissures, which display an increase in mitochondrial density within 12 h of injury due to increased axonal transport and translocation into growth cones (Han et al., 2016). Axon regeneration of mammalian neurons also appears to involve axonal mitochondrial transport, as loss of function in the mammalian-specific mitochondrial anchor protein syntaphilin results in enhanced axon regeneration (Zhou et al., 2016). Furthermore, the mammalian-specific transport protein Armcx1 is required for the enhanced regenerative capacity of some retinal ganglion axons in a regeneration-enhanced background (Cartoni et al., 2016). In contrast, Rawson and colleagues reported that in *C. elegans ric-7* mutant worms, which cannot transport mitochondria into PLM axons, severed axons retained their regenerative competence (Rawson et al., 2014).

In addition to mitochondrial transport, studies in mammalian peripheral axons or in *Drosophila* have found that injury triggers mitochondrial fission (Chen et al., 2016; Kiryu-Seo et al., 2016). In contrast to our findings in PLM sensory neurons, the fission-defective mutant *drp-1* is defective in *C. elegans* motor commissure regrowth (Han et al., 2016), although it is unclear if this reflects a requirement for fission during the injury response or if it results from the chronic depletion of axonal mitochondria in these mutants. Taken together, these studies suggest the effects of injury on axonal mitochondrial distribution may vary depending on the species and neuronal subtype.

In PLM neurons, injury does not appear to trigger drastic remodeling of axonal mitochondria. Nonetheless, our data show that mitochondrial function is important in PLM axon regrowth. We tested over 20 genes with known or predicted roles in mitochondrial biogenesis or function, assessing mitochondrial transport, calcium uptake, mitophagy, the fission/fusion cycle, and the electron transport chain. We find no evidence that mitophagy on its own is essential for regrowth, as also reported for *C. elegans* motor neurons (Han et al., 2016). Adaptors for mitochondrial transport along microtubules also did not appear to play direct roles in PLM regrowth, although we have not excluded possible redundancy between the two *miro* genes. Axon regrowth was essentially normal in *drp-1* mutants (fission defective) or *fzo-1* mutants (fusion defective) implying that the fission/fusion cycle is not rate limiting to PLM axon regrowth. In contrast, *eat-3* (inner mitochondrial membrane fusion defective) mutants displayed significantly reduced regrowth, potentially reflecting an influence on the electron transport chain.

Our observations that loss of function in only five out of eleven ETC subunits resulted in significantly impaired axon regrowth support a more specific role for the electron transport chain in axon regrowth. Consistent with our findings, Han and colleagues also found that *isp-1* mutants are strongly defective in GABAergic motor neuron commissure regrowth (Han et al., 2016). However, in contrast, Han and colleagues found that *nuo-6* mutants are mildly defective in motor neuron regrowth, whereas we find *nuo-6* mutants show normal PLM regrowth. This suggests that GABAergic motor neurons may be more sensitive to lowered ETC function than are PLM neurons, or that different neuron types depend on different ETC components for regrowth.

A key question is why axon regrowth is sensitive to loss of function in some but not other components of the ETC. It should be noted that in addition to their best-known roles in ATP synthesis, mitochondrial function affects diverse aspects of cellular metabolism. Chronic disruption of electron transport, as in the mutants studied here, triggers complex cellular responses, including remodeling of metabolism to preserve energy production. Electron transport dysfunction can trigger the mitochondrial retrograde signaling pathway (Liu and Butow, 2006), the mitochondrial unfolded protein response (Nargund et al., 2012), as well as a host of other metabolic responses (Morgan et al., 2015) including aberrant NADH:NAD^+^ ratios (Falk et al., 2008) and altered one-carbon metabolism (Bao et al., 2016). A common result of ETC dysfunction is elevated production of mitochondrial ROS, which in some cases induces elevated levels of detoxifying enzymes. However, our data together with those of Han et al. (2016) argue against a role for mitochondrial calcium handling or ROS production in axon regeneration.

Focusing on the energetic phenotypes of the *C. elegans* mutants studied here, disruption of Complex I—as in *gas-1(fc21)* mutants—impairs Complex I activity, but animals compensate by elevating Complex II function and organismal ATP levels are normal (Kayser et al., 2004). Conversely, loss of Complex II function—as in *mev-1(kn1)* mutants—leads to a compensatory increase in Complex I function resulting in overall normal ATP levels (Braeckman et al., 2000; Senoo-Matsuda et al., 2001). Defects in ubiquinone synthesis, such as in *clk-1* mutants, strongly reduce Complex I-dependent respiration, but overall metabolism and ATP content are normal, presumably due to compensatory upregulation of glycolytic pathways for ATP production (Braeckman et al., 1999). Complex III-impaired *isp-1* mutants display the most dramatic axon regrowth defects in our assay and have been reported to have either normal (Yang and Hekimi, 2010a) or reduced (Yee et al., 2014) ATP levels. Conversely, *rad-8* mutants have normal ATP levels (Braeckman et al., 2000), yet display reduced regrowth. ATP levels have not been examined in *nduf-2.2* or *nduf-7* mutants. *nuo-6* mutants, which had normal PLM regrowth, have been reported to have either elevated (Yang and Hekimi, 2010a) or reduced (Yee et al., 2014) ATP levels. Thus, there is so far no clear correlation between respiratory chain outputs and regeneration, although it should be noted that whole organism measurements of ATP or other metabolites may not necessarily extend to individual neurons.

In seeking possible commonalities among the subset of mitochondria- and ETC-related genes required for axon regeneration, we note that several genes have human orthologs implicated in hereditary optic neuropathies, wherein retinal ganglion cells or their axons degenerate, leading to blindness. The *eat-3* ortholog *OPA1* is well known for its association with dominant optic atrophy, an inherited condition characterized by retinal ganglion cell degeneration (Lenaers et al., 2012). Mutations in *NDUFS2* and *NDUFS7*, orthologs of *nduf-2.2/gas-1* and *nduf-7*, respectively, are associated with mitochondrial Complex I deficiency, which can cause hereditary optic neuropathy (Triepels et al., 1999; Bugiani et al., 2004; Tuppen et al., 2010). Human mutations in *UQCRFS1*, the human ortholog of *isp-1*, the Complex III Rieske iron-sulfur protein, have not been directly associated with human disease, but Complex III deficiency in general is linked to optic neuropathy (Benit et al., 2009). A recent report found that mutations in *RTN4IP1*, the *rad-8* ortholog, lead to inherited optic neuropathy (Angebault et al., 2015). Although many of the other genes screened in this study have human orthologs associated with a variety of diseases, including degenerative diseases affecting the nervous system, none are currently associated with optic neuropathies. Speculatively, this suggests that *C. elegans* sensory neurons and human retinal ganglion cells share similarities in their dependence on specific components of the ETC, perhaps for metabolic or other homeostatic purposes. The finding that inhibition of the mitoUPR in the *nduf-2.2* mutant background could restore axon regeneration to wild type levels presents the possibility that specific cellular adaptations and signaling cascades remain active in this subset of ETC mutants.

An additional common feature among four of the six genes identified here as being required in axon regrowth is their biochemical function as iron-sulfur proteins, the key electron donors and acceptors in the ETC. Why these proteins are specifically required while other components of the same ETC complexes are not required is unclear. One possibility is that these proteins, due to their interface with electrons, experience a high rate of structural damage during respiration such that their degradation and replacement is rate-limiting. Another possibility is that under conditions of high respiratory demand—stresses such as axon injury—dysfunction of iron-sulfur proteins might lead to release of labile iron, leading to further cellular damage or the initiation of processes such as ferroptosis (Dixon and Stockwell, 2014; Yang and Stockwell, 2016). In support of both of these options, we found that overexpression of the iron-sulfur proteins NDUF-2.2 and ISP-1 can enhance regrowth, which may provide a ready pool of replacement proteins or might sequester labile iron, or both. It remains unclear why overexpression of the NDUF-2.2 paralog GAS-1 did not enhance regrowth; perhaps NDUF-2.2 plays a more dominant or specific role in neurons, while GAS-1 has a larger role in non-neuronal tissues. Together these data suggest that ETC iron-sulfur proteins play a specialized role in neurons and in axon regeneration.

Our finding that we could genetically suppress *rad-8* growth phenotypes with either of the *clk-1* mutant alleles was a serendipitous observation during double mutant construction. It is possible that loss of *clk-1* function leads to mild stress and upregulation of stress response pathways that might suppress the *rad-8* defects. However, other ETC mutants—which may also activate stress response pathways—did not similarly suppress *rad-8(mn163)* phenotypes in the viable double-mutants, in some cases (*nduf-2.2, isp-1)* actually exacerbating the slow growth of *rad-8* mutants. Moreover, although both *clk-1* alleles suppressed *rad-8* growth rate defects, only the point mutant (*e2519*) and not the null allele suppressed the *rad-8(mn163)* regeneration defect, suggesting the *e2519* allele might possess altered function, as has been noted (Branicky et al., 2006). Similarly, RAD-8 may also has tissue-specific functions. It was notable that restoration of RAD-8 expression only in neurons also rescued organismal growth rates (this study and Fujii et al., 2011), while expression of the RAD-8ΔMLS construct in neurons strongly impaired overall growth rates. These findings support a link between neuronal mitochondria and overall animal growth rates (Ndegwa and Lemire, 2004; Berendzen et al., 2016), although overall growth rates and axon regeneration after injury are not themselves correlated. Future studies on the molecular mechanisms of these ETC-related genes may help shed further light on the role that mitochondria play in the axon regenerative program and may, through homology with human genes, provide insights into human optic atrophies.

## Author contributions

WK: Designed and performed experiments, analyzed data, and wrote the paper. TH: Designed and performed experiments, analyzed data, and commented the paper. ZW: Performed experiments. AC and YJ: Designed experiments, analyzed data, and wrote the paper.

### Conflict of interest statement

The authors declare that the research was conducted in the absence of any commercial or financial relationships that could be construed as a potential conflict of interest.

## Supplementary material

Figure S1 **Mitochondria in the worm nervous system. (A)** Brightfield and fluorescence images of wild type worms expressing mitoGFP under the pan-neuronal *rgef-1* promoter. Zoomed in views of the **(A')** head and **(A”)** tail ganglia are outlined. **(B)** Expression of mitoGFP in the PLM neuron using the *mec-4* promoter. **(C)** The density of axonal mitochondrial puncta in the first ~100 μm from the cell body in the PLM neuron remains stable from the final larval stage (L4) through the seventh day of adulthood (A7). *p*-values calculated using Student's *t*-test vs. L4: ns, not significant.

Figure S2 **Mutants in cristae shape-related genes have normal axon regeneration**. Single mutant analysis of genes linked to cristae formation or shape show no significant defects in axon regeneration. *p*-values calculated using Student's *t*-test vs. same day control: ns, not significant.

Table S1 **Mutant strains and genotyping primer sequences used**.

Table S2 **Plasmids and cloning primer sequences used**.

Table S3 **Transgenic array strain information**.

Table S4 **Genotyping primer sequences used for strains with transgenic arrays**.
