# Supplementary figures and images for: A Select Subset of Electron Transport Chain Genes Associated with Optic Atrophy Link Mitochondria to Axon Regeneration in Caenorhabditis elegans
Source: Front Neurosci. 2017 May 10;11:263. doi: 10.3389/fnins.2017.00263 (PMC5423972; doi:10.3389/fnins.2017.00263)

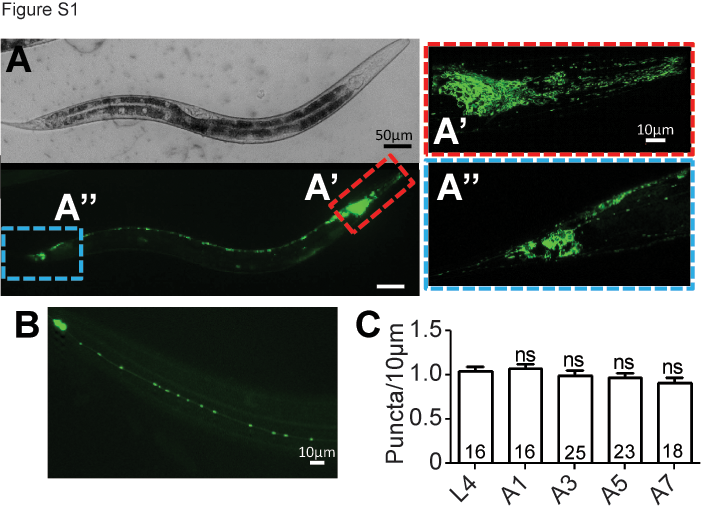

Supplement: Figure S1 — Mitochondria in the worm nervous system. (A) Brightfield and fluorescence images of wild type worms expressing mitoGFP under the pan-neuronal rgef-1 promoter. Zoomed in views of the (A') head and (A”) tail ganglia are outlined. (B) Expression of mitoGFP in the PLM neuron using the mec-4 promoter. (C) The density of axonal mitochondrial puncta in the first ~100 μm from the cell body in the PLM neuron remains stable from the final larval stage (L4) through the seventh day of adulthood (A7). p-values calculated using Student's t-test vs. L4: ns, not significant. [file Image1.tif]

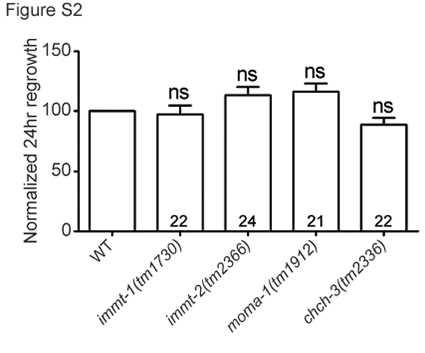

Supplement: Figure S2 — Mutants in cristae shape-related genes have normal axon regeneration. Single mutant analysis of genes linked to cristae formation or shape show no significant defects in axon regeneration. p-values calculated using Student's t-test vs. same day control: ns, not significant. [file Image2.tif]
