# Supplementary material for: A Select Subset of Electron Transport Chain Genes Associated with Optic Atrophy Link Mitochondria to Axon Regeneration in Caenorhabditis elegans
Source: Front Neurosci. 2017 May 10;11:263. doi: 10.3389/fnins.2017.00263 (PMC5423972; doi:10.3389/fnins.2017.00263)
Supplement: Table S1 — Mutant strains and genotyping primer sequences used. [file Table1.pdf]

Table S1: Mutant strains used in this study

Strain	Genotype	Forward Primer	Reverse Primer	Internal Primer
CZ10175	*Pmec-4::GFP(zdIs5)*	n/a	n/a	
CZ13419	*Pmec-4:mcherry(juIs252); Pmec-4::mitoGFP(juEx3328)*	n/a	n/a	
CZ24522	*Prgef-1::mitoGFP(juEx7517)*	n/a	n/a	
CZ20394	*zdIs5; miro-1(tm1966)*	GCTCAACGATTTCCAGAAGC	TCTTCAGCGTTTCCATCAGA	
CZ21813	*zdIs5; miro-2(tm2933)*	TGTCGTCTACTCGGTTACAG	GGTGTTGCAAACCAGCTAGA	CCCTTCGGAGAGTACTGGCTA
CZ20973	*zdIs5 trak-1(tm1572)*	ACGAGTGGAGGGAAGATAGGATTAG	AGACGCAAGGGAGTCATAGAGAG	
CZ21242	*zdIs5; mcu-1(ju1154)*	TCGTCACTGAAATGGCAAAA	AGCTAACGGGAAGATGCTG	AAATCCTGCCCACATGACTC
CZ22052	*zdIs5; emre-1(tm6230)*	TTTTTGGCTTTAGGCTGTTGA	CAGGCATTAAGCATATTTCCAA	GGGACAAAGACCATTCAC
CZ20972	*zdIs5; pink-1(tm1779)*	CGCTGAAGGCTCGGATGATG	CGACCGTGGCGAGTTACAAG	
CZ13540	*zdIs5; pink-1(ok3538)*	CACACTGCACGTTGGTATGA	AATTCCATTGATTCCATCCG	
CZ11438	*zdIs5; pdr-1(gk448)*	ACAAAAACATGGGGCTTCAA	CTGGGATCTTCCATCGTCAT	
CZ21240	*zdIs5; pdr-1(tm395)*	ACAAAAACATGGGGCTTCAA	CTGGGATCTTCCATCGTCAT	
CZ21241	*zdIs5; pdr-1(tm598)*	ACAAAAACATGGGGCTTCAA	CTGGGATCTTCCATCGTCAT	
CZ18815	*zdIs5; drp-1(tm1108)*	GTTGTCTCCCTCTCCCTGGT	GGGAAACGAATCATCTCCTG	
CZ25169	*zdIs5; fis-1(tm1867)*	TGTTGGATTTCGATTGTGGA	GGTTCGGAGAAGAGCATTGA	
CZ25139	*zdIs5; fis-1(tm2227)*	GGTGTCCCGAGAGAATCAGA	GAGCATGCGTTCAAGGTACA	
CZ20703	*zdIs5; fis-2(gk363)*	ATCTATGGCGCGAATGAGAC	GTCATCGCTTTTTGTGAGCA	
CZ20672	*zdIs5; fis-2(gk414)*	ATCTATGGCGCGAATGAGAC	GTCATCGCTTTTTGTGAGCA	
CZ21029	*zdIs5; fis-2(tm1832)*	ATCTATGGCGCGAATGAGAC	GTCATCGCTTTTTGTGAGCA	
CZ24628	*zdIs5; fzo-1(tm1133)*	GTGGACTTCACAGCTGTATG	CGAATTCTGGAGAAAGATCG	
CZ25440	*zdIs5; eat-3(ad426)*	CGATTCGTCATACAACACC	GGAAATAGCTTTCCTTCAAG	
CZ22823	*zdIs5; eat-3(tm1107)*	CGATTCGTCATACAACACC	GGAAATAGCTTTCCTTCAAG	
CZ25320	*zdIs5; chch-3(tm2336)*	GAAGTGGTGCGCATTGACC	CACTTCAAAACACGTCCTGG	
CZ25230	*zdIs5; immt-1(tm1730)*	GTTCCGCCAGTGAAACAAGT	AATCTCGCTTTCCATCGTTC	
CZ25231	*zdIs5; moma-1(tm1912)*	ACCGCTTGATTTTCCAGATG	AATGCCCACCCATAATGCT	
CZ25232	*zdIs5; immt-2(tm2366)*	CGCTGTTAGAACGCCAGATT	TGCCTTCAGATGAGCAACAG	AGAAGAAGCCAGTCGCTGAG
CZ18152	*zdIs5; gas-1(fc21)*	TTTGCAGGCTCTCCCATACTTC	GACTCCATGTTCTCCTTCATCT	
CZ12422	*zdIs5; nduf-2.2(ok437)*	TTCAGACTGGCGGAACTCTT	ACACCATCCTCTTCGTCCAC	CGAAATACGGGAGTGCCTAC
CZ24563	*zdIs5 nduf-7(et19)*	TTGCCGTATCTAATGCAACAAC	AAAATGAATGAAGTGGGGCTTA	
CZ18154	*zdIs5 nuo-6(qm200)*	CGTCGTGAATACGATCCTCA	TCTCTCGAAAGTCAGGCACA	
CZ22888	*zdIs5; mev-1(kn1)*	ATTCACAGTCCGAGGCAAAG	GAAAATGATGGGGAAAGCAA	
CZ22825	*zdIs5 sdha-2(tm1420)*	CTCCGAAGGTGGAATGAAAA	TACCTGGCAATCGTTGTTGA	
CZ21219	*zdIs5; clk-1(e2519)*	TTCAGTGTGGCTGCTTATGCTC	ATTAACTTGCCGTGCTCGTG	
CZ10964	*zdIs5; clk-1(qm30)*	TTCAGTGTGGCTGCTTATGCTC	ATTAACTTGCCGTGCTCGTG	
CZ22068	*zdIs5 rad-8(mn163)*	AGTGCCGCTTCCAGTCATAA	CGTTGTGAGACCCTGGCTAT	
CZ10965	*zdIs5; isp-1(qm150)*	TCACCAATGGACTTGCTGTC	ACGTCCAGAAGCGTCGTAGT	
CZ22890	*zdIs5; ucr-2.3(ok3073)*	TCAATTGGTGAGTCGCATTC	TTGCGAGCAAATAAACACCA	
CZ22887	*zdIs5; ucr-2.3(pk732)*	TCAATTGGTGAGTCGCATTC	TTGCGAGCAAATAAACACCA	
CZ21962	*zdIs5; asg-2(ok3344)*	TTTGAGCATTAGAGTGAGTTTTTG	CCAGTAGGCATAGTGGGGTG	
CZ21961	*zdIs5; asg-2(tm1472)*	TCCTCGCGTTTTCACAATTT	TCTTGTATCCTCCGGTCTGG	ACAGGCGGGAGTATCGATTT
CZ9221	*zdIs5; egl-19(ad695)*	ACTAGAAATATGGTTTGCCCTC	TTCTCCACGGCATTCTGCTTC	
CZ12951	*zdIs5; isp-1(qm150); egl-19(ad695)*	see above	see above	
CZ15571	*zdIs5; Pmec-4::dlk-1(juSi50)*	GTCCTCCGACTTCTCTACAG	GCCATTCAAGTTCGGAGATAG	
CZ19270	*zdIs5; isp-1(qm150); Pmec-4::dlk-1(juSi50)*	see above	see above	
CZ24018	*zdIs5 rad-8(mn163); nduf-2.2(ok437)*	see above	see above	
CZ24025	*zdIs5 rad-8(mn163); isp-1(qm150)*	see above	see above	
CZ24027	*zdIs5 rad-8(mn163); ucr-2.3(pk732)*	see above	see above	
CZ23900	*zdIs5 rad-8(mn163); clk-1(qm30)*	see above	see above	
CZ24026	*zdIs5 rad-8(mn163);clk-1(e2519)*	see above	see above	
CZ22053	*zdIs5; atfs-1(tm4525)*	CATAATGGTTCGCTGCAAAA	CCAAAATTGGAGAAATCATGC	
CZ25656	*zdIs5; nduf-2.2(ok437); atfs-1(tm4525)*	see above	see above	
CZ22070	*zdIs5; ztfs-1(tm4919)*	CATAATGGTTCGCTGCAAAA	CCAAAATTGGAGAAATCATGC	
CZ25657	*zdIs5; nduf-2.2(ok437); atfs-1(tm4919)*	see above	see above	
