# Supplementary material for: A Select Subset of Electron Transport Chain Genes Associated with Optic Atrophy Link Mitochondria to Axon Regeneration in Caenorhabditis elegans
Source: Front Neurosci. 2017 May 10;11:263. doi: 10.3389/fnins.2017.00263 (PMC5423972; doi:10.3389/fnins.2017.00263)
Supplement: Table S2 — Plasmids and cloning primer sequences used. [file Table2.pdf]

Table S2: Rescuing array plasmids and cloning primers

Plasmid	Contains	Forward Primer	Reverse Primer
pCZGY1873	pCR8-isp-1	ATGGCTTCTCTTGCTAGATC	CTACAGTAACATGGTATCT
pCZGY1874	Prgef-1::isp-1	n/a	n/a
pCZGY2926	pCR8-nduf-2.2	GAGATGCTTTCGCGATCATT	CGAACCAACCGTACATTCAA
pCZGY2929	Pdpy-30::nduf-2.2	n/a	n/a
pCZGY2954	pCR8-gas-1	AACAATGTTGGGTAGAAAGATCG	TGTTAGCTCTATTTTTATTGTTGTGAG
pCZGY2961	Prgef-1::gas-1	n/a	n/a
pCZGY2935	pCR8-rad-8	TTCAGCAAAATGATCGAAAAA	CTTGCGGCAAAGTGTTTCTT
pCZGY2940	Prgef-1::rad-8	n/a	n/a
