# Supplementary material for: A Select Subset of Electron Transport Chain Genes Associated with Optic Atrophy Link Mitochondria to Axon Regeneration in Caenorhabditis elegans
Source: Front Neurosci. 2017 May 10;11:263. doi: 10.3389/fnins.2017.00263 (PMC5423972; doi:10.3389/fnins.2017.00263)
Supplement: Table S3 — Transgenic array strain information. [file Table3.pdf]

**Table S3: Transgenic array strains**

| Strain | Transgene | Plasmid | Concentration | Coinjection marker |
|---|---|---|---|---|
| CZ24522 | *Prgef-1::mitoGFP* | pCZGY2256 | 25ng/µl | Pgcy-8::mKate2 |
| CZ13419 | *Pmec-4::mitoGFP(juEx3328)* | pCZGY1876 | 25ng/µl | Pttx-3::GFP |
| CZ25197 | *zdIs5; isp-1(qm150); Prgef-1::isp-1(juEx4406)* | pCZGY1874 | 25ng/µl | Pttx-3::RFP |
| CZ25138 | *zdIs5; isp-1(qm150); Prgef-1::isp-1(juEx5711)* | pCZGY1874 | 20ng/µl | Pttx-3::RFP |
| CZ23759 | *zdIs5; nduf-2.2(ok437); Pdpy-30::nduf-2.2(juEx7197)* | pCZGY2929 | 25ng/µl | Pttx-3::RFP |
| CZ24892 | *zdIs5; nduf-2.2(ok437); Pdpy-30::nduf-2.2(juEx7589)* | pCZGY2929 | 20ng/µl | Punc-122::RFP |
| CZ24929 | *zdIs5; gas-1(fc21); Prgef-1::gas-1(juEx7499)* | pCZGY2961 | 20ng/µl | Pttx-3::RFP |
| CZ24905 | *zdIs5; gas-1(fc21); Prgef-1::gas-1(juEx7500)* | pCZGY2961 | 20ng/µl | Pttx-3::RFP |
| CZ23722 | *zdIs5 rad-8(mn163); Prgef-1::rad-8(juEx7154)* | pCZGY2940 | 25ng/µl | Pttx-3::RFP |
| CZ23723 | *zdIs5 rad-8(mn163); Prgef-1::rad-8(juEx7155)* | pCZGY2940 | 25ng/µl | Pttx-3::RFP |
| CZ23520 | *zdIs5; Pdpy-30::nduf-2.2(juEx7197)* | pCZGY2929 | 25ng/µl | Pttx-3::RFP |
| CZ24769 | *zdIs5; Pdpy-30::nduf-2.2(juEx7589)* | pCZGY2929 | 20ng/µl | Punc-122::RFP |
| CZ18149 | *zdIs5; Prgef-1::isp-1(juEx4406)* | pCZGY1874 | 25ng/µl | Pttx-3::RFP |
| CZ19009 | *zdIs5; Prgef-1::isp-1(juEx5711)* | pCZGY1874 | 20ng/µl | Pttx-3::RFP |
| CZ24489 | *zdIs5; Prgef-1::gas-1(juEx7498)* | pCZGY2961 | 20ng/µl | Pttx-3::RFP |
| CZ24490 | *zdIs5; Prgef-1::gas-1(juEx7499)* | pCZGY2961 | 20ng/µl | Pttx-3::RFP |
| CZ23730 | *zdIs5; Prgef-1::rad-8(juEx7154)* | pCZGY2940 | 25ng/µl | Pttx-3::RFP |
| CZ23731 | *zdIs5; Prgef-1::rad-8(juEx7155)* | pCZGY2940 | 25ng/µl | Pttx-3::RFP |
