# Supplementary material for: A Select Subset of Electron Transport Chain Genes Associated with Optic Atrophy Link Mitochondria to Axon Regeneration in Caenorhabditis elegans
Source: Front Neurosci. 2017 May 10;11:263. doi: 10.3389/fnins.2017.00263 (PMC5423972; doi:10.3389/fnins.2017.00263)
Supplement: Table S4 — Genotyping primer sequences used for strains with transgenic arrays. [file Table4.pdf]

Table S4: Primers for genotyping rescued strains

Strain	Transgene	Forward primer	Reverse primer
CZ25197	*zdIs5; isp-1(qm150); Prgef-1::isp-1(juEx4406)*	AACGTCGTGCTCTTCCAACT	GAGCTTGCTGTGTCATGTTCTC
CZ25138	*zdIs5; isp-1(qm150); Prgef-1::isp-1(juEx5711)*		
CZ23759	*zdIs5; nduf-2.2(ok437); Pdpy-30::nduf-2.2(juEx7197)*	TTATCCGTGCTCCTGGTTTT	ACACCATCCTCTTCGTCCAC
CZ24892	*zdIs5; nduf-2.2(ok437); Pdpy-30::nduf-2.2(juEx7589)*		
CZ24929	*zdIs5; gas-1(fc21); Prgef-1::gas-1(juEx7499)*	TCAATTGACCACAACGAAAAA	GACTCCATGTTCTCCTTCATCT
CZ24905	*zdIs5; gas-1(fc21); Prgef-1::gas-1(juEx7500)*		
CZ23722	*zdIs5 rad-8(mn163); Prgef-1::rad-8(juEx7154)*	TCTCGTTGTCCAACAAGTGG	CGTTGTGAGACCCTGGCTAT
CZ23723	*zdIs5 rad-8(mn163); Prgef-1::rad-8(juEx7155)*		
